# Mesenchymal stem cell-based therapy for female stress urinary incontinence 

**DOI:** 10.3389/fcell.2023.1007703

**Published:** 2023-01-13

**Authors:** Xiaochun Liu, Tingting Li, Jia Zhang, Xiling Lin, Wenzhen Wang, Xiaodong Fan, Lili Wang

**Affiliations:** ^1^ Third Hospital of Shanxi Medical University, Shanxi Bethune Hospital, Shanxi Academy of Medical Sciences, Tongji Shanxi Hospital, Taiyuan, China; ^2^ Key Laboratory of Cellular Physiology at Shanxi Medical University, Ministry of Education, and the Department of Physiology, Shanxi Medical University, Taiyuan, China; ^3^ School of Public Health, Shanxi Medical University, Taiyuan, China; ^4^ School of Biomedical Engineering at Taiyuan University of Technology, Taiyuan, China

**Keywords:** mesenchymal stem cells, stress urinary incontinence, stem cell-based therapy, animal model, mechanisms of action

## Abstract

Stress urinary incontinence (SUI) adversely affects the quality of life of patients, while the currently available surgical and non-surgical therapies are not effective in all patients. Application of mesenchymal stem cells (MSCs) for regaining the ability to control urination has attracted interest. Herein, we reviewed the literature and analyzed recent studies on MSC-based therapies for SUI, summarized recent treatment strategies and their underlying mechanisms of action, while assessing their safety, effectiveness, and prospects. In addition, we traced and sorted the root literature and, from an experimental design perspective, divided the obtained results into four categories namely single MSC type therapy for SUI, MSC-based combination therapy for SUI, treatment of SUI with the MSC secretome, and other factors influencing MSC therapy. Although evidence demonstrates that the treatment strategies are safe and effective, the underlying mechanisms of action remain nebulous, hence more clinical trials are warranted. Therefore, future studies should focus on designing clinical trials of MSC-based therapies to determine the indications for treatment, cell dosage, appropriate surgical strategies, and optimal cell sources, and develop clinically relevant animal models to elucidate the molecular mechanisms underlying stem cell therapies improvement of SUI.

## 1 Introduction

**FIGURE 1:**
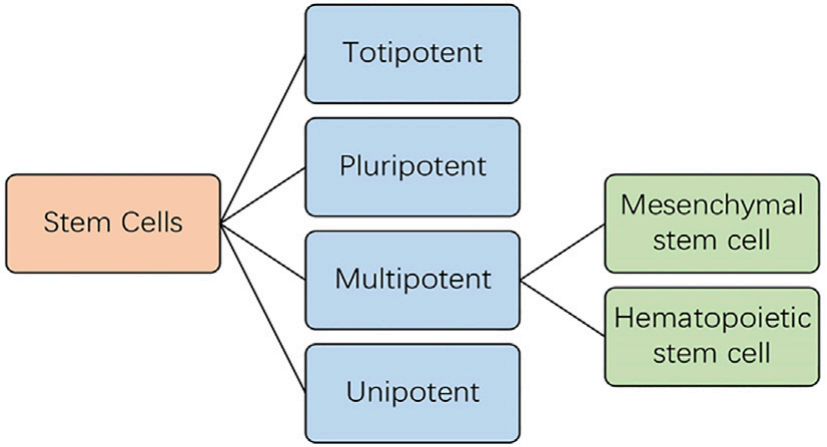
Stem cells are classified according to their developmental stages.

Stress urinary incontinence (SUI) refers to the leakage of urine during sneezing, laughing, coughing, and exercise, due to a sharp increase in intra-abdominal pressure (Hart et al., 2015). Although SUI occurs in both males and females, females are more affected (Abufaraj et al., 2021). This review focuses on female SUI. SUI affects the quality of life of females and increases the financial burden of individuals and health systems. The incidence of SUI in China, South Korea, and Nepal is 18.9% (Zhang et al., 2015), 33.6% (Shim et al., 2021), 24.1% (Chen et al., 2020), respectively. Initial treatment usually includes pelvic floor muscle training and behavioral intervention. Furthermore, bladder training, urethral inserts, and vaginal devices may also alleviate SUI. Injections of urethral bulking agents also alleviate leakage. Adipose tissue, especially MFAT (lipogems and nanofat), is common urethral bulking agents in the treatment of stress urinary incontinence in female patients. Compared with non-surgical treatments, surgical intervention provides better treatment outcome against SUI. Pubovaginal sling and colposuspension are the most effective surgical strategies (Nygaard and Heit, 2004; Silwal Gautam et al., 2014). However, surgical procedures are associated with various adverse effects. The current treatment modalities are suboptimal, as they can only alleviate the symptoms to a certain degree, without curing SUI. In addition, the incidence of urinary incontinence may increase with age, hence other treatment strategies remain warranted. With advancements in regenerative medicine, stem cells have become a focal point in life sciences research owing to their self-renewal and multidirectional differentiation properties. Stem cells are be classified as either embryonic or adult stem cells, according to their developmental stages. Adult stem cells have been previously studied; however, ethical considerations have hampered the extensive study of embryonic stem cells. Based on their differentiative potential, stem cells are divided into totipotent, pluripotent, multipotent and unipotent (Sobhani et al., 2017), of which multipotent stem cells are the current focus of research and are further categorized into hematopoietic and mesenchymal stem cells (MSCs) (Mishra et al., 2020; Figure 1). MSCs, originate from the mesoderm and ectoderm and usually reside in connective tissue and organ stroma (Zheng et al., 2021), exhibit high proliferation, self-renewal, and multidirectional differentiation properties, and display immune-regulatory, paracrine, and other functions (Dominici et al., 2006). MSCs are easily obtained from multiple sources, including the bone marrow, peripheral fat, umbilical cord blood or placenta, 64 amniotic fluid, gingiva, olfactory mucosa, and urine (Shin et al., 2020). MSCs home into injured sites and differentiate into corresponding cells, thereby replacing injured cells, in addition to secreting neurotrophic factors. Additionally, MSCs promote tissue repair by secreting bioactive factors, such as extracellular vesicle (EVs) (Cheng et al., 2020; Zheng et al., 2021), which are lipid-bound secreted by cells into the extracellular space (Zaborowski et al., 2015; Zheng et al., 2021). The three essential EV subtypes are exosomes, micro-vesicle, and apoptotic bodies, classified according to their biogenesis, release pathways, size, content, and function (Borges et al., 2013; Yanez-Mo et al., 2015; Zaborowski et al., 2015). At present, research has primarily focused on the former two. MSC- derived EVs bear varying types of molecules involved in intercellular communication, immune response, homeostasis, coagulation, inflammation, angiogenesis, and antigen presentation (Harrell et al., 2019; Zhao et al., 2020).

Stem cells have been extensively used in medicine; the multilineage differentiation potential and immune modulatory properties of MSCs make them a promising treatment option for various clinical conditions. Previous phase III trials have provided evidence for the effectiveness and safety of MSCs. MSCs have been used in the treatment and prevention of graft-versus-host disease (NCT03106662), mandible fractures (NCT02755922), severe coronavirus disease 2019 (NCT05122234), retinitis pigmentosa (NCT04224207), and inherited retinal dystrophy (NCT04224207). Therapeutic indications of stem cells for SUI are its high prevalence and chronic nature (Shin et al., 2020). Although the number of studies on MSC-based therapy for SUI is limited, such research is critical for addressing urinary control that affects the quality of life of many females globally. Here, we summarize and analyze relevant literature published over the past 10 years, outlining the mechanisms underlying MSC-based therapies in SUI treatment and the latest advances in this field.

## 2 Single MSC therapy for SUI

**FIGURE 2:**
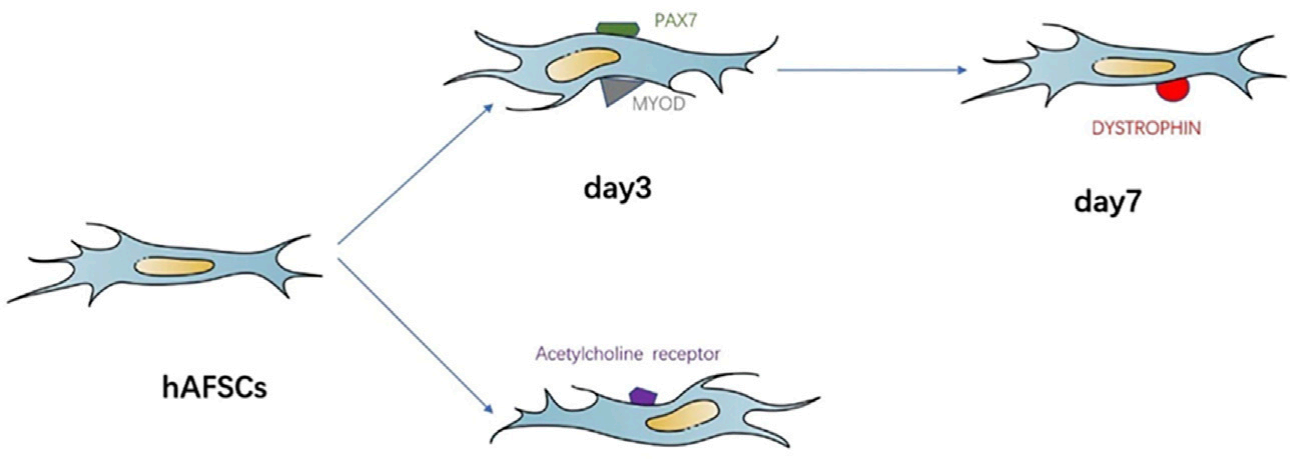
Simplified representation of the MSCs in myogenic differentiation and neurogenic differentiation.

The multidirectional differentiation potential of MSCs has been suggested to play a role in SUI treatment. Therefore, previous studies have used single MSCs to treat SUI in animal models to validate this hypothesis; Table 1 lists the features of various single MSC therapies for SUI. For example, Kim et al. (2012) investigated the effect of human amniotic fluid stem cells (hAFSCs) using an animal model with SUI that underwent bilateral pudendal nerve transection. The authors found that hAFSCs can differentiate into muscle progenitor cells when cultured in myogenic induction media, and therefore hypothesized that the grafted hAFSC underwent myogenic differentiation *in situ*, potentially inducing the regeneration of the host muscle. In addition, the level of neurogenic gene expression in animals with hAFSCs was substantially higher than in those without, suggesting that their injection can exert positive physiological effects on nerve regeneration and the re-formation neuromuscular junctions (Figure 2).

**TABLE 1:** Study characteristics dealing with Single type of MSC therapy for stress urinary incontinence.

Author/year	Type of study	Stem cell source	Animal/model	Method of injection	Detection method	Main outcomes	Conclusion
Kim et al. (2012)	*In vivo*	Human amniotic fluid-derived	Mice/BPNT	Periurethral injection	LPP, CP, Histological, IHC, RT-qPCR	Expression of early myogenic differentiation markers decreased; expression of the middle and late differentiation markers increased; mean LPP and CP were increased; expression of neuronal markers and acetylcholine receptor is existing	The grafted hAFSCs might have undergone *in situ* myogenic differentiation and induced host muscle regeneration; Periurethral injection of hAFSCs into an SUI animal model restored the urethral sphincter to apparently normal histology and function
Silwal Gautam et al. (2014)	*In vivo*	Adipose-derived	Rabbit/injure the internal urethralorifice by spraying liquid nitrogen	Periurethral injection	LPP, IHC, Quantification of muscle area	LPP; reconstructed skeletal and smooth muscle are increased; myoglobin, SMA, and Pax7 antibodies; nerve cell markers, vascular endothelial cell marker; transforming growth factor β1, nerve growth factor, and vascular endothelial growth factor were positive	Implantation of autologous adipose-derived cells into the cryoinjured rabbit urethras promotes the recovery of urethral function
Li et al. (2016)	*In vivo*	Rats Adipose-derived	Rats/VD + OV	Urethral injection	Voiding function examine; WB; immunofluoresce nce; IHC staining	Both voiding function and histopathological structures are better recovered in MTs group; MTs express higher level of VEGF,TSG-6 *in vitro* and represent a higher retention rate *in vivo*	Urethral injection of MTs better restored the voiding function than ADSCs
Sadeghi et al. (2016)	*In vivo*	Human bone marrow-derived	Rats/VD	Periurethral injection	LPP; HE; trichrome; IHC	Connective tissue area/urethral section area proportion and vascular density were higher in the local hMSC-treated group; the ratio of vascular density to whole urethra area was significantly greater in the hMSC group	hMSC paracrine action can be a possible mechanism of SUI improvement rather than hMSC enraftment into tissues and *in situ* differentiation. mechanism of tissue repair or improvement orchestrated is by hMSCs or their secretory factors
Sadeghi et al. (2020)	*In vivo*	Human bone marrow-derived	Rats/VD	Periurethral injection	RNA-seq	DEGs were functionally associated with tissue repair, angiogenesis, neurogenesis and oxidative stress suppression; DEGs included a variety of cytokines, extracellular matrix stabilization; DEGs expressions are related to DNA damage repair, transcription activation, stem cell regulation, cell survival, apoptosis, self-renewal, cell proliferation, migration, and injury response	hBM-MSC exposed to the damaged urethra alter the tissue response to acute injury and improve tissue function through gene expression changes
Garcia-Arranz et al. (2020)	*In vivo*/prospective, non-randomize d phase I-IIa clinical trials	Human adipose-derived	/	endoscopic intraurethral injection	urodynamic studies; pad test, quality-of-life; incontinence questionnaires	Five women showed an objective improvement of >50% (*p* < .05) and a subjective improvement of 70%–80% from baseline	ASC therapy is a feasible and safe therapy for the treatment of urinary incontinence in men and women

**FIGURE 3:**
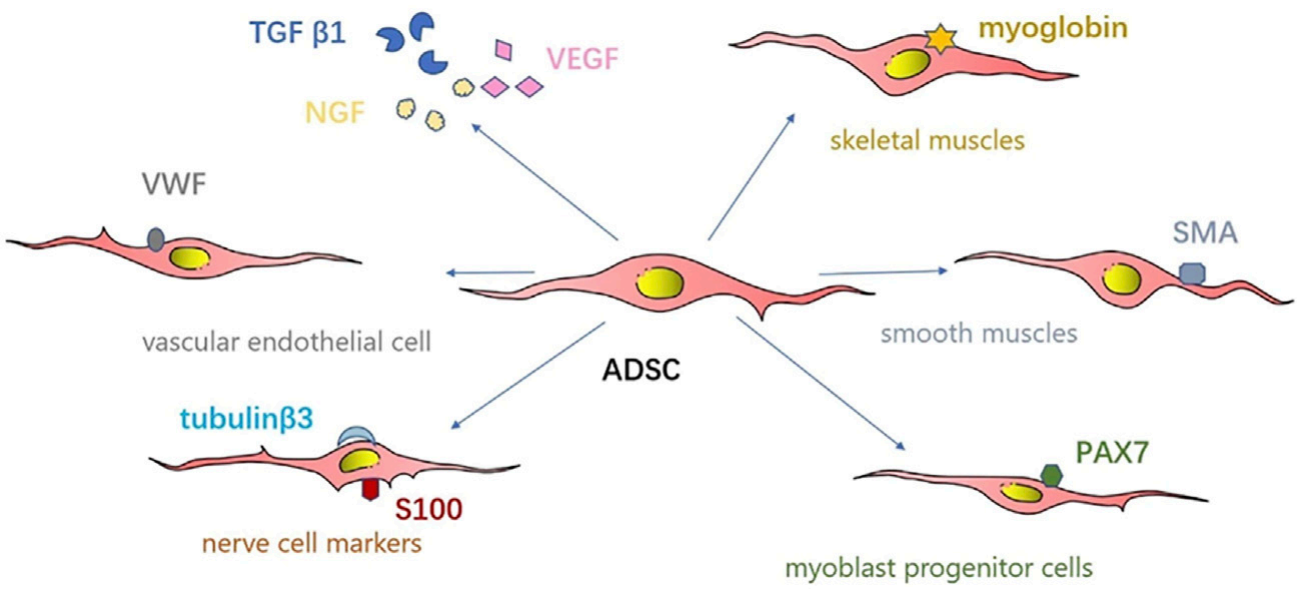
Simplified representation of the mechanism of ADSCs therapy.

In another study, Silwal Gautam et al., 2014 investigated the use of injected autologous adipose-derived stem cells (ADSCs) into cryoinjured rabbit urethras injured by spraying liquid nitrogen for 20 s to simulate SUI. Autologous ADSCs were transplanted 7 days post injury and urinary function was assessed 14 days later. The authors observed that the leak point pressure in the cell-implanted group was substantially higher than that in the cell-free control group. In addition, immunohistochemical examination demonstrated that the reconstructed skeletal and smooth muscle areas were significantly more developed in the cell-implanted regions than control regions. The implanted ADSCs were immunohistochemically positive for skeletal muscle, such as myoglobin, smooth muscle actin, and paired box 7 antibodies, smooth muscle, myoblast progenitor cells, and nerve cell markers (tubulin β3, calcium-binding protein P, vascular endothelial cell markers, and von Willebrand factor). In addition, some implanted cells were positive for the transforming growth factor β1, nerve growth factor, and vascular endothelial growth factor. The authors concluded that the autologous ADSC therapy for the cryoinjured rabbit urethras worked *via* a comprehensive effect involving myogenic differentiation, neuronal regeneration, neoangiogenesis, and bulking effects (Figure 3).

The adipose tissue is the most practical cell source used to treat female urinary incontinence. Li et al. (2016) sought to improve the efficacy of ADSCs to treat a SUI rat model that underwent vagina dilation followed by bilateral ovariectomy. The authors used the hanging drop method to transform ADSCs into microtissues (MTs), which showed better recovery in terms of voiding function and histopathological structure compared to the ADSC group. *In vitro*, the authors also found that MTs expressed higher levels of vascular endothelial growth factor and TNFα-stimulated genes/proteins, supporting the higher retention rates observed *in vivo* and concluded that the therapeutic effect of urethral injection of MTs was superior to that of ADSCs in SUI rats.

Although the feasibility of single MSC therapy for SUI has been recognized, the mechanism of actions has been explained in diverse ways. For example, Sadeghi et al. (2016) found no evidence to support the hypothesis that human MSCs (hMSCs) differentiated into muscle cells after injection in midurethral tissue samples of rats with SUI. Instead, the authors speculated that the paracrine action of hMSCs could be the underlying mechanism of SUI remission. Furthermore, the vascular density of the entire urethral area in the hMSCs-treated group was greater than that in the control group. This suggests that the mechanism of action of the hMSCs, or their secretory factors, involves the induction of vascular preservation or neovascularization in the acutely injured tissue. Interestingly, the study illustrated that the injured urethral tissue did not exhibit prominent inflammatory cell infiltration following hMSC injection, which differed from the findings of Grinnemo et al.’s (2006) of massive macrophage infiltration following hMSC transplantation. Reasons for the different outcomes might be attributed to the cell source, the culture environment *in vitro*, and the type of receptors. To test the hypothesis that long-term paracrine effects of hMSCs reduce inflammatory responses and modulate a healing microenvironment, Sadeghi et al. (2020) performed the first all-round transcriptomic analysis of hMSC therapy for postpartum SUI. The authors observed various differentially expressed genes in the injured urethra treated with hMSCs related to several signaling pathways involved in tissue regeneration and repair, such as wound healing, vasoprotection, angiogenesis, neurogenesis, and neuroprotection as well as oxidative stress inhibition. Data on the immunomodulatory mechanisms underlying MSC therapy for SUI are limited, hence this new resource provides momentous transcriptomic information on the interaction of hMSCs and injured tissue for identifying potential therapeutic targets for postpartum SUI therapy and prevention.

Two prospective, non-randomized phases I–II a clinical trials have reported that stem cell-based SUI therapy provides acceptable functional results with minimal complications or side effects in female patients (Garcia-Arranz et al., 2020). However, a small number of clinical trials have previously analyzed and compared the results, hence the overall data are not sufficient for assessing the long-term safety of the treatment.

## 3 MSC-based combination therapy

Although the feasibility of MSC therapy for SUI has been demonstrated (Kim et al., 2012; Silwal Gautam et al., 2014; Sadeghi et al., 2016; Garcia-Arranz et al., 2020; Sadeghi et al., 2020), MSCs alone may be insufficient to regenerate whole urethral components. Table 2 lists the features of MSC-based combination therapies for SUI. One limitation of MSC therapy is the low survival rate of MSCs in injured urethral tissue following local engraftment (Kean et al., 2013). Bioactive molecules or biomaterials could enhance the survival and integration of the transplanted cells (Hart et al., 2015). Intravenous injection of MSCs has been reported to lower survival rates since most of were trapped in the lungs and other organs (Acosta et al., 2015). MSCs express diverse unique chemokine receptors that are likely to play a key role in their engraftment (Honczarenko et al., 2006); among them, the chemokine/chemokine receptor axis, C-C motifligand 7 (CCL7)/C-C motif receptor 1 (CCR1), is involved in the recruitment process of MSCs in urethral and vaginal tissues following postpartum injury (Huang et al., 2010). Jiang et al. (2020) injected modified bone marrow-derived MSCs to overexpress CCR1 into SUI rats through the tail vein, along with 2 μg of the active CCL7 peptide around the urethra. The rats showed the highest rate of successfully transplanted MSCs in the injured tissue and level of functional recovery of SUI when compared with the control groups. However, the authors examined the transplantation rate and urethral recovery of MSCs only 1 week after the rats had been injured during childbirth, and therefore did not consider the long-term outcomes.

**TABLE 2:** Study characteristics dealing with MSC based combination therapy for stress urinary incontinence.

Autho r/year	Type of study	Stem cell source	Animal/model	Method of injection	Other components of the association	Detection method	Main outcomes	Conclusion
Jiang et al. (2020)	*In vivo*	Rats/Bone marrow-derived	Rats/VD +BOR	urethral injection and intrave	CCL7	EUS EMG; PNMBP; LPP	UI = MSC-CCRI + CCL7 group are the most engraftment of MSCs to the injured	Combined treatment with CCR1-overexpressing MSCs and CCL7 can increase engraftment of MSCs and promote the
Jalali Tehrani et al. (2021)	*In vivo*	Rats Adipose-derived	Rats/BP NT	transurethra l injection	muscle-derived stem cell	urodynamic assays; Immunocytochemic al analysis; Histopathological and IHC evaluations; UPP	The muscular layer of the external sphincter is regenerated; periurethral striated muscle layer is thickened; urethral pressure profile is significantly increased	The application of co-cultured adipose-derived and muscle-derived stem cells could be associated with higher therapeutic value in stress urinary incontinence patients compared with singular-cell treatments
Burdzinska et al. (2018a)	*In vivo*	Goats/Bone marrow-derived	Goats/ag ed multipar ous female goats	intraurethra l injections using an endoscope	muscle-derived stem cells	UPP measurements; immunohistochemi cal evaluation	DID-derived signal was strongest; striated muscle formation; MUCP and FA were increased	MDC-MSC co-transplantation provides a greater chance of improvement in urethral closure than transplantation of each population alone
Kuismanen et al. (2014)	*In vivo*	Human ADSCs	/	Transurethr al injection	Bovine collagen gel; saline	Cough test; Validated questionaires	1 of 5 patients displayed a negative cough test 6 months later. 3 of 5 patients showed negative cough test 1 year later. All five patients have subjective improvement	The treatment with autologous ASCs is safe and well tolerated
Zhang et al. (2018)	*In vivo*	Rats/VD +BOR	Rats/VD +BOR	Novel nanoyarn biomaterial	Sling surgery	LPP; histological tests	The cell-laden nanoyarn maintained LPP at normal levels for 8 weeks, while LPP in the nanoyarn alone group gradually decreased after surgery	Nanoyarn has sufficient efficacy on maintaining LPP in UI rat model. Moreover, the combination of ADSCs and a nanoyarn scaffold bring a better therapeutic effect
Wang et al. (2017)	*In vivo*	Rats ADSCs	/	/	Polyglycolic acid fibers (PGA)	Maximal load Young’s modulus; tissue and collagen structures	The engineered neo-sling tissue that generated using ADSCs lead a significant improvement in biomechanical properties	ADSCs may serve as a novel cell source for tissue sling engineering and could improve treatment for patients with SUI.
Du et al. (2013)	*In vivo*	Rats/Bone marrow-derived	Rats/BP NT	Inject into the urethra and bladder neck submucosal ly	muscle-like cells, calcium alginate composite gel	LPP; HE staining	Growth of blood vessels increased; BMSC, and muscle-like cells gathered around the new blood vessels; muscle-like cells grew into elongated spindle-like cells; Desmin, and α-SMA staining were positive expression	Compound of BMSC, muscle-like cells, and calcium alginate composite gel has the potential to differentiate into muscle cells in the microenvironment of SUI rat model. the increase in urethral resistance is associated with the volume effect of calcium alginate gel
Jin et al. (2016a)	*In vitro* and *in vivo*	Rats/Bone marrow-derived	Rats/VD	Local injection	bFGF from PLGA NPs	13.1.1 PCR; WB; ELISA; Consciou s cytometry; LPP	*In vitro*, the elastin-expressing BM-MSCs produce more collagen and elastin; *In vivo*, the outcome of urodynamic tests is improved	Transplantation of elastin-expressing BMSCs into PFD rats could significantly alleviate the symptoms of PFD. This beneficial effect could be further enhanced by sustained released of bFGF from PLGA NPs
Jin et al. (2016b)	*In vitro* and *in vivo*	Human/Bone marrow-derived	Rats/VD	Local injection	13.1.2 bFGF-loaded PLGA NPs	13.1.3 PCR; WB; ELISA; MTT assay; CMG test; LPP	The secretion of elastin of BM-MSCs is increase; the urodynamic test results is improved	When injected together with the bFGF-loaded PLGA NPs, genetically engineered miR-29a-3p-inhibited BMSCs significantly improved results of urodynamic tests

Previous studies have attempted to combine MSCs from two different sources to enhance their efficacy of SUI treatment. For instance, Jalali Tehrani et al., 2021 used muscle-derived stem cells (MDSCs), ADSCs, and a combination of the two types of stem cells to treat an animal SUI model that underwent bilateral pudendal nerve transection surgery. The authors found that the urethral pressure curve in the group with the combined two types of stem cells was higher than that of the remaining groups, and showed a thicker periurethral striated muscle layer than those of the groups with only one type of stem cell. The authors suggested that it is beneficial for the recovery of the urethral sphincter function to locally inject stem cells into the urethra and that the therapeutic outcome is amplified when combining two types of stem cells, and finally hypothesized that the application of co-cultured stem cells could enhance therapeutic effects due to the more comprehensive environment of cytokines and paracrine factors.

Similar results have been reported by Burdzinska et al., 2018a, who conducted a study on aged multiparous female goats and found that the co-transplantation of MSCs and MDSCs was more effective than cell therapy alone. However, the underlying mechanism of the increased efficacy of MSC-MDSC co-engraftment remains unclear. The authors concluded that MDSCs enhanced the myogenic activity of MSCs, and that the latter increased the survival rate of the former *via* paracrine action. The results are consistent with their previous report on soluble factors secreted by MSCs that enhanced the fusion index of myoblasts (Kulesza et al., 2016).

In recent years, significant progress has been made in the field of tissue engineering, particularly in seed cells, scaffold materials, and organization building. Kuismanen et al. (2014) have assessed the effectiveness and safety of autologous adipose stem cells (ASCs) in treating female stress urinary incontinence and, using the cough test, found that following treatment of 5 SUI patients with ASCs combined with bovine collagen gel and saline, one patient tested negative 6 months later, while and three tested negative after 1 year. Besides, the authors also observed some subjective improvement in all five patients. This was the first study to describe the use of autologous ASCs in combination with collagen gel for female SUI treatment and illustrate the safety and well-tolerance of the autologous ASCs-based therapy.

Calcium alginate gel is a mature tissue-engineering scaffolding material. Du et al. (2013) demonstrated that calcium alginate composite gels, bone marrow-derived mesenchymal stem cells (BMSCs), and muscle-like cells could differentiate into muscle cells in an SUI rat model. Hematoxylin-eosin staining showed that desmin, a cytoskeletal intermediate-filament protein and α-skeletal muscle actin were expressed in the tissue, suggesting the regeneration of smooth and striated muscle cells. In the authors assessed urethral function and identified a strong relationship between an increase in urethral resistance and volume effects of calcium alginate gel.

Furthermore, factors such as optimal alginate gel porosity, optimal combination ratio of gel, muscle-like cells, and stem cells are closely associated with cell proliferation and muscle fiber formation (Du et al., 2013). Overall, transplanting seed cells and scaffold material is a promising therapeutic strategy whose safety and efficacy have been demonstrated. Non-etheless, long term studies are lacking, albeit necessary, to obtain evidence to support MSC-based combination therapy.

To limit adverse foreign body reactions induced by non-absorbable materials, previous studies have attempted to use combination of mesenchymal stem cells and biomaterials for SUI to enhance the biocompatibility of materials. Among them, adipose tissue is the most commonly used source of mesenchymal stem cells and its safety and therapeutic effect have also been verified. Zhang et al. (2018) have developed a new nanoyarn biomaterial *via* a dynamic liquid electrospinning system to apply nanoyarn as a urinary sling. The authors combined nanoyarn with adipose-derived stem cells (ADSCs) and observed that the LPP in the nanoyarn alone group gradually descended, while the cell-laden nanoyarn maintained LPP at normal levels for 8 weeks. The authors suggested that combination of ADSCs and a nanoyarn scaffold is effective to maintain normal LPP in urinary incontinence rat model, which provides a potentially novel strategy to treat female SUI. The implantation of suburethral sling is an effective therapy for SUI. Wang et al. (2017) have attempted to overcome the inherent limitations of suburethral sling implantation by seeding ADSCs used in treating female stress urinary incontinence on polyglycolic acid (PGA) fibers, which generated a neo-scaffold with a shape mimicking a sling complex. The engineered neo-sling tissue displayed a significant improvement in biomechanical properties, which increased the treatment efficacy against SUI.

SUI is a type of pelvic floor dysfunction (PFD), characterized by connective tissue abnormalities and alterations of elastin metabolism. In a rat model, Jin et al., 2016a studied the effects of stem cell-based, elastin gene-modified, and nanoparticle-mediated multidisciplinary therapy against pelvic floor tissues under PFD. Elastin-overexpressing BMSCs were transplanted into damaged tissue induced after vaginal distension. The intervention had beneficial therapeutic effects in PFD rats, where poly lactic-co- glycolic acid nanoparticles were first loaded, followed by loading of basic fibroblast growth factor to enable continuous release of the former, which enhanced the therapeutic effects of BMSCs with modified elastin gene.

Subsequently, to study other latent regulatory factors, such as microRNAs(miRs), Jin et al., 2016b broadened the scope of the therapeutic notion. miRs are a set of non-coding single-stranded RNA molecules encoded by endogenous genes with a length of approximately 22 nucleotides involved in the regulation of post-transcriptional gene expression in animals and plants. miRs can negatively regulate messenger RNAs (mRNAs) by distinguishing specific complementary sequences primarily located in the 3′-UTR of the target mRNAs, thereby either inhibiting its translation or degrading it (Ott et al., 2011). For example, the miR-29 family of miRs downregulates the expression of elastin (Sudo et al., 2015; Zhang et al., 2015). More precisely, miR-29a-3p downregulates the endogenous expression of elastin in BMSCs (Jin et al., 2016a). The inhibition of miR-29a-3p in BMSCs markedly increases the expression and secretion of elastin, consequently enhancing the therapeutic effect of BMSCs following injection into PFD rats.

Finally, although previous studies have indicated that the effects of MSCs combined with bioactivemolecules, other cell types, MSC biomaterials, or MSCs genetically modified for the treatment of SUI are superior to therapies based solely on MSCs, the cost-benefit relationships of such combinatorial therapies requires further assessment.

## 4 Treatment of SUI based on the MSC secretome

With recent interest in the exploitation of the paracrine effects of MSCs for SUI treatment, the MSC secretome has been increasingly analyzed, mainly in three aspects: concentrated conditioned media (CCM), exosomes, and EVs, which is also called MSC-based cell-free product. Table 3 outlines the features of SUI treatments based on the MSC secretome previously reported; Dissaranan et al. (2014) hypothesized that MSCs can home into the pelvic organs of animal models following childbirth injury, facilitating the recovery of SUI exerted by paracrine factors. To test this hypothesis, the author first demonstrated the ability of MSCs to home into affected pelvic organs of SUI rat models and validated the effectiveness of MSCs and CCM in SUI treatment by injecting MSCs intravenously, and CCM, into the periurethral tissue. The authors observed an increase in elastin near the external urethral sphincter and the formation of smooth muscles of the urethra, suggesting that MSCs can home into the vagina and urethra, and deduced that the synthesis of elastin was increased *via* secretion of paracrine factors, which, in turn, promoted SUI recovery (Dissaranan et al., 2014). Similarly, Deng et al. (2015) demonstrated the ability of MSCs to repair damaged tissue in dual injuries of muscles and nerves *via* secretion of bioactive factors. Other studies have shown that the feasibility of CCM in SUI treatment is similar to that of MSCs (Jiang et al., 2021; Zhuang et al., 2021).

**TABLE 3:** Study characteristics treating stress urinary incontinence by MSC secretome.

Autho r/year	Type of study	Stem cell source	Animal/model	Method of injection	Detection method	Main outcomes	Conclusion
Dissaranan et al. (2014)	*In vivo*	Bone marrow-derived	Rats/VD	IV/periurethra l	LPP; EUS EMG; *Ex Vivo* Fluorescent Imaging; Immunofluorescent Staining; Histology 13.1.4	Smooth muscle layers and urethral function improved in rats treated with MSCs or CCM	MSCs delivered IV facilitate recovery of the urethra after simulated childbirth injury, likely *via* secreting paracrine factors. local application of these paracrine factors stimulating an equivalent recovery to MSC treatment
Deng et al. (2015)	*In vivo*	Rat bone marrow-derived	Rats/PNC + VD	IV/IP injection	13.1.5 LPP; PNSBP	Urethral function, urethral elastogenesis and pudendal nerve regeneration improved in rats treated with MSCs or CCM	MSCs act *via* their secretions in this dual muscle and nerve injury.
Jiang et al. (2021)	*In vitro* and *in vivo*	Bone-marrow derived	Rats/VD	Co-culture/Periurethral injection	CCK-8 assays; cell migration assay; WB; CR; RNA-seq; Detection and verification of the JAK2/STAT4 pathway; LPP; Histology, IHC, and IF	Differentially expressed genes were enriched for cell adhesion, extracellular fibril organization and angiogenesis; ALPP is increased; increased numbers of fibroblasts, improved collagen fibers arrangement and elevated collagens content in the AVW of rats receiving BMSC-CCM	BMSC secretome activates AVW fibroblasts and contributes to the functional and anatomic recovery of simulated birth trauma-induced SUI in rats
Zhuang et al., 2021	*In vitro* and *in vivo*	Human pluripotent stem cell-derived	Rats/transabdo minal urethrolysis	13.1.6 peri-urethral injection	LPP, PCR, WB, Elastin staining, qualitative examination of elastin morphology, IF, Gelatin zymography	pSMC-CM upregulated MMP-2, TIMP-2, Collagen, and elastin gene expression, improved urethral pressure and increased collagen and elastin expression	Conditioned media from smooth muscle cell progenitors derived from human pluripotent stem cells improved urethral leak point pressure and collagen and elastin content in the SUI rat
Liu et al. (2018)	*In vitro*	Human adipose-derived	Vaginal fibroblasts from women with SUI	Co-culture	13.1.7 RT-PCR; WB	ADSC-exos upregulate the expression of the col1a1, TIMP-1 and TIMP-3 in fibroblasts, downregulate the expression of MMP-1 and MMP-2	ADSC-exos increased type I collagen contents by increasing collagen synthesis and decreasing collagen degradation in vaginal fibroblasts from women with SUI
Li et al. (2021)	*In vivo*	Bone-marrow derived	Rats/transabdo minal urethrolysis	injectionaroun d the pubococcygeus muscle	IF, PCR, WB, CCK-8 assay, HE staining and Masson staining ALPP, MBV, estimated marginal mean	SIRT1/exos improved ALPP, MBV, and estimated marginal mean in rats of SUI; enhanced the proliferation, differentiation and activation of SCs; exhibited their positive effect on BM-MSCs by activating the ERK signaling	SIRT1/exos meliorated pubococcygeus muscle injury in rats by promoting ERK pathway
Wang et al. (2021)	*In vivo*	Adipose-derived	Rats/VD	Co-culture	13.1.8 Immunofluore scence Staining 13.1.9; PCR; WB; Dual-Luciferase Reporter Gene Assay	ADSCs-EVs secrete miR-93 to regulate the ECM remodeling of fibroblasts and promote the activation of SCs; overexpression of F3 reverse the effects of ADSCs-EVs on ECM remodeling of SUI primary fibroblasts and SC activation	miR-93 delivered by ADSCs-EVs could regulate ECM remodeling of SUI fibroblasts and promote activation of satellite cells through targeting F3
Zhang et al., 2020	*In vitro* and *in vivo*	Bone-marrow derived	Rats/transabd ominal urethrolysis/Primary fibroblasts	trans-urethral wall injecton	WB, LPP, HE staining, IF, sEV miRNA sequencing, Dual-luciferase reporter assay, Cell transfection	MMP-2, TIMP-2, Collagen, elastin and MMP-9 activity increased; urethral function improved; BM-MSCs-EVs miR-328a-3p can be transferred from BM-MSC to fibroblasts and regulate the Sirt7/TGF-β1 signaling pathway	BMMSC-sEV miR-328a-3p represses Sirt7 expression to promote ECM remodeling of damaged urethral sphincters by activating the TGF-β1 signaling pathway

**FIGURE 4:**
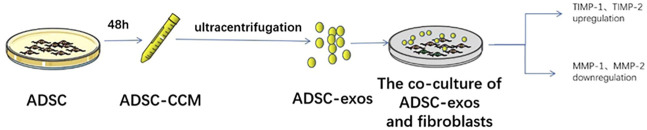
Simplified representation of the effect of ADSC-exos on fibroblasts.

To elucidate the precise mechanism of action of CCM in SUI treatment, previous studies have conducted more in-depth analysis. For instance, Liu et al. (2018) demonstrated that exosomes extracted from ADSC-CCM can regulate the metabolism of type I collagen in fibroblasts and observed that the expression levels of matrix metallopeptidase 1 (MMP1) and MMP2 in fibroblasts decreased, while those of metallopeptidase inhibitors one and three increased, when the fibroblasts were treated with exosomes secreted by ADSCs. The MMP gene family encodes different types of collagenases, enzymes that degrade collagen. Conversely, metallopeptidase inhibitors are specific inhibitors of MMPs that protect collagen against degradation. Therefore, the authors also concluded that exosomes secreted by ADSCs promote the synthesis of collagen while inhibiting its degradation, thereby enhancing the support of the periurethral tissues to the urethra (Liu et al., 2018). Exploring the active ingredients in MSC-CCM provides a theoretical basis for understanding the molecular mechanisms underlying SUI treatment with MSCs (Figure 4).

**FIGURE 5:**
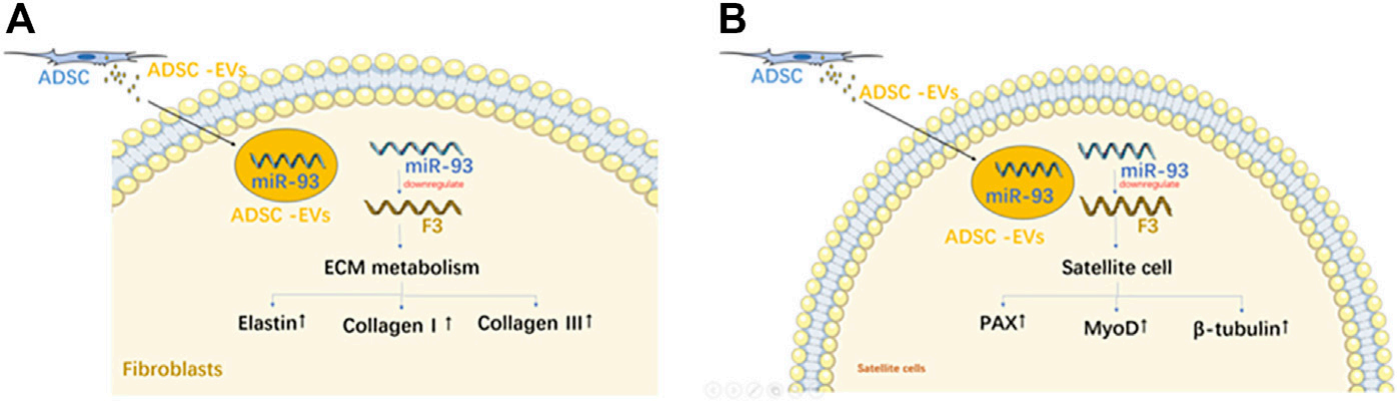
The mechanism of EVs from ADSC on fibroblasts **(A)**; The mechanism of EVs from ADSC on satellite cell **(B)**.

**FIGURE 6:**
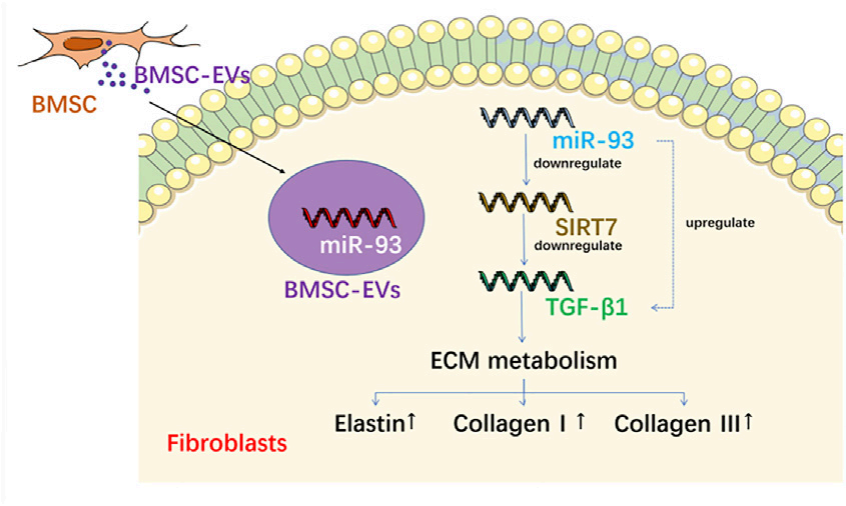
The mechanism of EVs from BM-MSC on fibroblasts.

Exosomes isolated from silent mating type information regulation 2 homolog 1-overexpressing BMSCs improved symptoms in SUI rats (Li et al., 2021), and enhanced the proliferation, differentiation, and activation of stem cells. Wang et al. (2021) demonstrated that EVs from ADSCs protected against SUI through the miR-93/F3 axis. EVs secreted by ADSCs could send miR-93 to fibroblasts and downregulate the expression of coagulation factor III, thereby remolding the extracellular matrix of SUI fibroblasts. A higher expression of miR-93 was also observed in SUI primary satellite cells induced by ADSC-EVs, which directly targeted the expression of coagulation factor III and upregulated the expression of Pax7 and myogenic differentiation (Figure 5). A similar pattern was observed by Zhang et al. (2020) who found that miR-328a-3p from BMSC-derived EVs was transferred to fibroblasts and regulated the extra cellular matrix metabolism *via* the sirtuin 7/transforming growth factor β1 signaling pathway (Figure 6).

## 5 Other factors influencing MSC therapy

Other key factors in the stem cell field include the cell source, injection site, dose, and type of technology used. Table 4 lists aspects associated with MSC-based SUI therapy. Cui et al. (2018) directly injected MDSCs and ADSCs into the bladder and urethra of SUI rats undergone bilateral pudendal nerve transaction. Then, 0-, 15-, 30-, and 60-day post MSC implantation, the urinary voiding function was assessed using a urine dynamics detector, which showed that MDSCs andADSCs improved urination in rats with intrinsic sphincter deficiency, while the effect of MDSC-treatment was more pronounced than in the ADSC-treated groups. Myosin and α-smooth muscleactin contents in the MDSC-treated groups tended to increase. The authors found that the efficacy of MDSCs was higher than that of ADSCs. Burdzinska et al., 2018b studied the limited precision with which cell suspensions are delivered from different injection sites to the target tissues, and performed transurethral and periurethral injections of autologous MSCs in female goats and analyzed differences in their distribution in the urethra. In the periurethral group, cells located in the urethral wall accounted for 68.7% of the total number ofinjected cells, and 67.0% in the transurethral group; in addition, the average proportions of cells in the external urethral sphincter were 18.8% and 17.0%, respectively. In the transurethral injection group, the authors observed leakage of cell suspension and concluded that the precision of delivery of cells into the external sphincter of the urethra was limited regardless of the injection method used. The low efficiency of cell delivery to the external urethral sphincter and the high leakage of cell suspension are key factors that limit the effectiveness of cell therapy (Burdzinska et al., 2018a). Janssen et al. (2019) determined whether increasing the number of MSC could enhance the efficacy of stem cell therapy by injecting MSCs into SUI rat models intravenously on days 1, 7, and 14 post injury. The results showed that all dosages improved urethral integrity by restoring urethral connective tissue and neuromuscular structure. However, two and three doses of MSCs significantly improved the leak point pressure, compared with single dose. This confirms that the efficacy of MSC in the treatment of postpartum urinary incontinence and SUI is enhanced *via* multiple doses.

**TABLE 4:** Characteristics of the study of MSC therapy for SUI in other aspects.

Author/year	Type of study	Stem cell source	Animal/model	Method of injection	Detection method	Main outcomes	Conclusion
Cui et al. (2018)	*In vivo*	Muscle-derived Adipose-derived	Rat/BPNT	Bladder-neck and transurethral sphincter injection	X-Gal Staining, HE stain; immunostaining; RT-qPCR; WB; urodynamic Test; Cell Tracking	MDSCs and ADSC improved the function of urination, myosin and α-SMA content	MDSCs and ADSCs have obvious effects in the treatment and/or prevention of ISD and transplantation of MDSCs is more effective than ADSC
Burdzinska et al. (2018b)	*In vivo*	Bone-marrow derived	white landrace goats/urethral profilometry	Periurethral injection or transurethral injection	UPP, isualization of cell depots in the urethras, IHC	The mean percentage of depots located in the urethral wall in relation to all performed injections amounted to 68.7% and 67% for PERI and RTANS groups, respectively. The mean proportions of depots which were identified in EUS amounted 18.8% and 17.1%, respectively. Suspension leakage was observed in 19% of transurethral injections	Accurate injections into EUS are rare. Higher number of injections increase the chance of precise delivery— performing eight injections per individual gives very high probability that at least part of a graft will be appropriately located
Janssen et al	*In vivo*	Bone-marrow derived	Rats/VD + PN C	IV	ULPP, EUS EMG, pudendal nerve electroneurography	Two and three dose of MSCs improve LPP, while a single dose of that not. Single, as well as repeated, MSC improve urethral integrity. MSC improve elastogenesis, prevented disruption of the external urethral sphincter, and enhanced pudendal nerve morphology	MSC therapy for postpartum incontinence and SUI can be enhanced with multiple doses
Jager et al. (2020)	*In vivo*	Human bone-marrow derived	Porcine cadaveric tissue	Trans-urothelium and connective tissue close to the sphincter muscle	Determination of the injection pressure required	the waterjet technology facilitated fast and precise injections of viable cells through urothelial, mucosal and submucosal layers to reach the sphincter muscle	Needle-free waterjet injections deliver cells in the urethra faster and more precisely when compared with needle injections without compromising their viability

Recently, Jager et al., 2020 investigated whether a needle-free waterjet apparatus could deliver living cells under defined settings (including injection pressure, delivery volume, transportation media, and fluid velocity). The authors attempted to accurately penetrate the urothelium and connective tissue adjacent to the sphincter muscle without penetrating the latter. Waterjet injections of MSCs into isotonic cell culture medium resulted in similar or more viable cells compared with those after needle injections. By controlling the injection pressure, waterjet injections of MSCs to the urethra are faster and more accurate than needle injections, without adversely affecting cell viability Jager et al. (2020). Our study enriches existing injection technologies and will facilitate related future research.

## 6 Conclusion

The strategies for MSC-based SUI therapies mainly include single MSC type therapy for SUI, MSC- based combination therapy, and MSC secretome-based therapy. Previous studies have explored the optimal sources of MSCs, most suitable injection sites, dosages, and administration methods, and have demonstrated how MSCs treat SUI in terms of muscle repair and regeneration, regulation of extracellular matrix metabolism, nerve regeneration, and blood supply, which provided insights into the molecular mechanisms underlying MSC- mediated SUI treatment. In addition, the limitations of the cited studies could guide future research. Research on the potential mechanisms of action of MSC therapy for SUI in nerve regeneration and immunomodulation is currently limited. Existing studies have included small sample sizes, with animal models majorly based on small rodents, and rarely on large primates, which creates uncertainty in generalizing and applying the derived conclusions to humans. Primate models can better reflect the pathophysiology of human SUI. Only few clinical trials have been conducted, with a small number of participants and short follow-up time, therefore the long-term efficacy of MSC- based therapy for SUI remains to be evaluated. Combining MSCs with other components or approaches in SUI treatment, although promising, presents additional obstacles that deserve further study. Although multidisciplinary approaches are particularly suited for exploiting the therapeutic potential of MSCs, and their treatment strategies are promising, it is also necessary to assess their associated costs. In addition, multicenter clinical trials are warranted to extend the follow-up time and expand the number of participants to improve methodology.

In addition, the emerging MSC-based cell-free therapy has shown great superiority and broad prospects in animal experiments, however, the extraction process of MSC- based cell-free products is complex, while the amount of extraction is the most important challenge for cell-free therapy to overcome. As more MSC-based cell-free therapies are explored, MSC-based cell-free therapies will achieve new breakthroughs and have significant clinical implication.

## Abbreviations

ADSC, adipose-derived stem cell; BMDSC, bone merrow-derived stem cell; CCM, concentrated conditioned media; EV, extracellular vesicle; hAFSC, human amniotic fluid stem cell; hMSC, human mesenchymal stem cell; MDC, muscle-derived stem cell; miR, microRNA; MMP, matrix metallopeptidase; MSC, mesenchymal stem cell; MV, microvesicle; PFD, pelvic floor dysfunction; SUI, stress urinary incontinence.
