# Supplementary material for: Mesenchymal stem cell-based therapy for female stress urinary incontinence
Source: Front Cell Dev Biol. 2023 Jan 13;11:1007703. doi: 10.3389/fcell.2023.1007703 (PMC9880261; doi:10.3389/fcell.2023.1007703)
Supplement: Supplementary file 1 [file Image5.pdf]

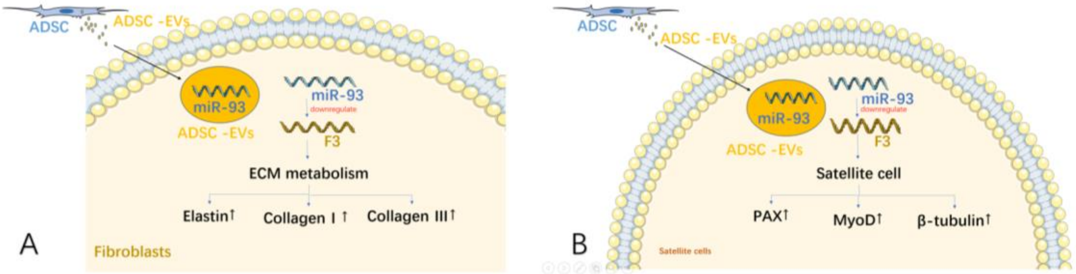

Fig. 5 The mechanism of EVs from ADSC on fibroblasts**(A)**; The mechanism of EVs from ADSC on satellite cell**(B)**
