# Supplementary figures and images for: Mesenchymal stem cell-based therapy for female stress urinary incontinence
Source: Front Cell Dev Biol. 2023 Jan 13;11:1007703. doi: 10.3389/fcell.2023.1007703 (PMC9880261; doi:10.3389/fcell.2023.1007703)

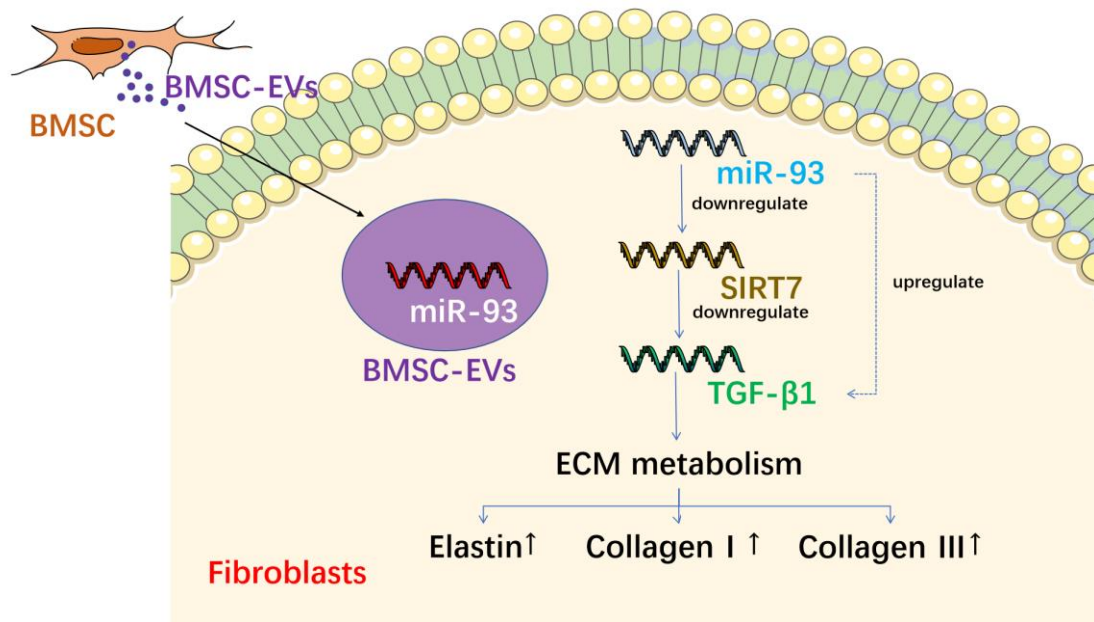

Fig. 6 The mechanism of EVs from BM-MSC on fibroblasts

Supplement: Supplementary file 2 [file Image6.pdf]

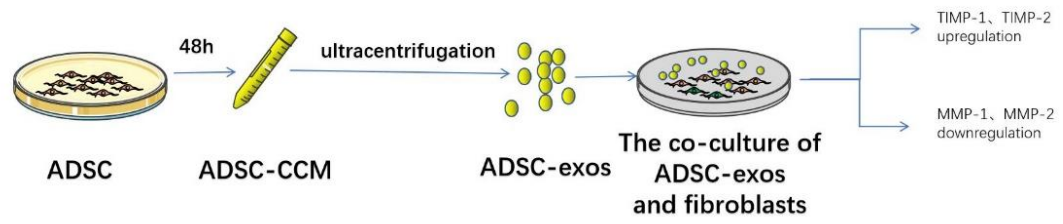

Fig. 4 Simplified representation of the effect of ADSC-exos on fibroblasts

Supplement: Supplementary file 3 [file Image4.pdf]

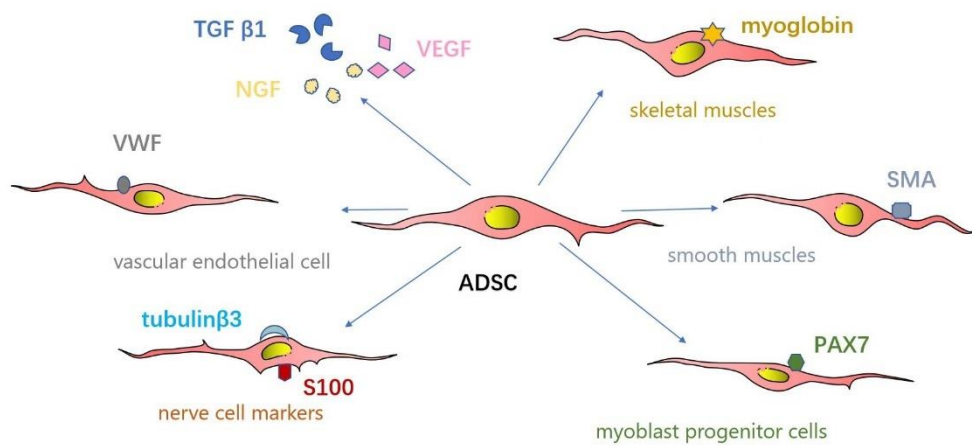

Fig. 3 Simplified representation of the mechanism of ADSCs therapy

Supplement: Supplementary file 5 [file Image3.pdf]

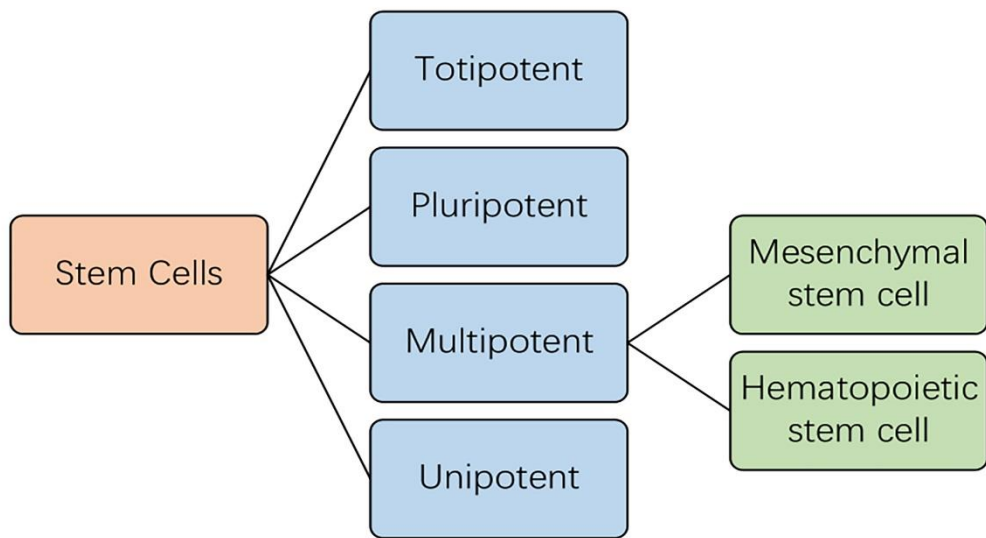

Fig. 1 Stem cells is classified according to their developmental stages

Supplement: Supplementary file 6 [file Image1.pdf]
