# Supplementary material for: Mesenchymal stem cell-based therapy for female stress urinary incontinence
Source: Front Cell Dev Biol. 2023 Jan 13;11:1007703. doi: 10.3389/fcell.2023.1007703 (PMC9880261; doi:10.3389/fcell.2023.1007703)
Supplement: Supplementary file 4 [file Image2.pdf]

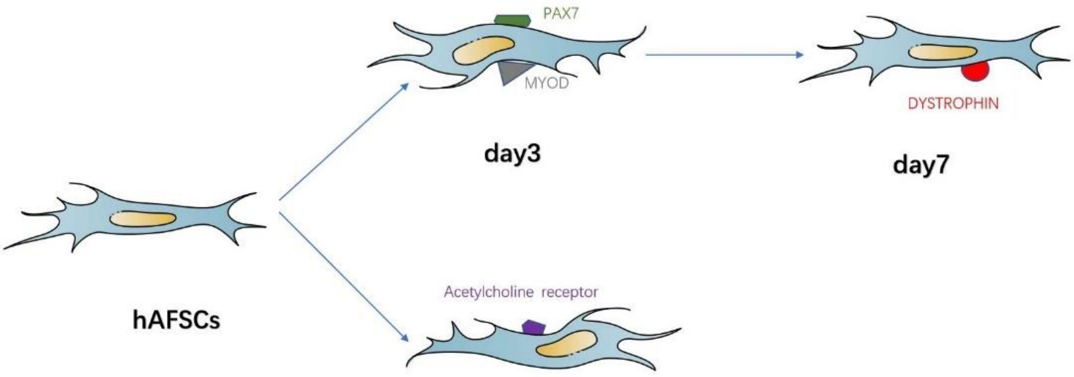

Fig. 2 Simplified representation of the MSCs in myogenic differentiation and neurogenic differentiation
